# A White Shark Equilibrium Optimizer with a Hybrid Deep-Learning-Based Cybersecurity Solution for a Smart City Environment

**DOI:** 10.3390/s23177370

**Published:** 2023-08-24

**Authors:** Latifah Almuqren, Sumayh S. Aljameel, Hamed Alqahtani, Saud S. Alotaibi, Manar Ahmed Hamza, Ahmed S. Salama

**Affiliations:** 1 Department of Information Systems, College of Computer and Information Sciences, Princess Nourah Bint Abdulrahman University, P.O. Box 84428, Riyadh 11671, Saudi Arabia; 2 SAUDI ARAMCO Cybersecurity Chair, Computer Science Department, College of Computer Science and Information Technology, Imam Abdulrahman Bin Faisal University, P.O. Box 1982, Dammam 31441, Saudi Arabia; 3 Department of Information Systems, College of Computer Science, Unit of Cybersecurity, King Khalid University, Abha 61421, Saudi Arabia; 4 Department of Information Systems, College of Computing and Information System, Umm Al-Qura University, Mecca 24382, Saudi Arabia; 5 Department of Computer and Self Development, Preparatory Year Deanship, Prince Sattam Bin Abdulaziz University, Al-Kharj 16278, Saudi Arabia; 6 Department of Electrical Engineering, Faculty of Engineering & Technology, Future University in Egypt, New Cairo 11845, Egypt; a.salama@fue.edu.eg

**Keywords:** smart grids, DDoS attacks, cybersecurity, feature selection, deep autoencoder, smart cities

## Abstract

Smart grids (SGs) play a vital role in the smart city environment, which exploits digital technology, communication systems, and automation for effectively managing electricity generation, distribution, and consumption. SGs are a fundamental module of smart cities that purpose to leverage technology and data for enhancing the life quality for citizens and optimize resource consumption. The biggest challenge in dealing with SGs and smart cities is the potential for cyberattacks comprising Distributed Denial of Service (DDoS) attacks. DDoS attacks involve overwhelming a system with a huge volume of traffic, causing disruptions and potentially leading to service outages. Mitigating and detecting DDoS attacks in SGs is of great significance to ensuring their stability and reliability. Therefore, this study develops a new White Shark Equilibrium Optimizer with a Hybrid Deep-Learning-based Cybersecurity Solution (WSEO-HDLCS) technique for a Smart City Environment. The goal of the WSEO-HDLCS technique is to recognize the presence of DDoS attacks, in order to ensure cybersecurity. In the presented WSEO-HDLCS technique, the high-dimensionality data problem can be resolved by the use of WSEO-based feature selection (WSEO-FS) approach. In addition, the WSEO-HDLCS technique employs a stacked deep autoencoder (SDAE) model for DDoS attack detection. Moreover, the gravitational search algorithm (GSA) is utilized for the optimal selection of the hyperparameters related to the SDAE model. The simulation outcome of the WSEO-HDLCS system is validated on the CICIDS-2017 dataset. The widespread simulation values highlighted the promising outcome of the WSEO-HDLCS methodology over existing methods.

## 1. Introduction

Smart grids (SGs) are an evolving technology, which provides intelligent monitoring, inters connectivity of multiple modes of generation, two-way data transmission, and improved resource utilization [1]. By raising the number of connected devices, it is tedious for the SG to access the distributed network. Therefore, the Internet of Things (IoT) is being used in the energy sector to enable bidirectional data transmission [2]. It involves the deployment of sensors, actuators, Radio-frequency Identification (RFID), and microcontrollers for communication and computation, to accomplish a two-way communication process [3]. If IoT is combined with SGs, it creates a widespread network of a cyber-physical system, which can be used to monitor and control connected devices remotely. Several countries have already implemented this technology, but approaches to implementation might differ based on the goals and policies of a country [4,5].

The interconnection of several devices from the domestic to the commercial level creates a communication network in the SGs. The physical component includes highly predictable, less technical, and few challenging issues, because of tedious human access and organized maintenance intervening with the faults instigated by material and equipment damage. At the same time, the challenging issues posed by the cyber network are highly complex, recurrent, and less predictable. Therefore, cyber-security has been regarded as a major power industry security target [6]. Cyber security in SGs is needed, as the embedded and general-purpose systems linked to it should be secure from cyber-attacks. Utilities need to ensure that cybersecurity in SGs for preserving the massive data flow and control signals indispensable to the SG for reaping the operational benefits derived from its implementation [7]. As SGs are a critical national infrastructure, cybersecurity in SGs should manage every possible threat from user errors and equipment failures.

Intrusion Detection is a technique for detecting attacks before or after they attain access to a security network. Integrating this method as to gateway is the fastest manner to combine it [8]. Deep Learning (DL), data mining, Machine Learning (ML), fuzzy logic (FL), evolutionary techniques, and other related approaches are comprised in Artificial Intelligence (AI). ML has become increasingly significant to researchers for risk recognition [9]. Researchers have utilized ML techniques, namely neural networks (NNs), support vector machines (SVMs), and random forests (RFs), for identifying jamming attacks. Researchers have used the ML approach for detecting social engineering attacks [7]. This method employs unsupervised learning; hence, it does not need that used for cyber-attacks in order to detect them.

This study develops a new White Shark Equilibrium Optimizer with a Hybrid Deep-Learning-based Cybersecurity Solution (WSEO-HDLCS) technique for a Smart City Environment. The goal of the WSEO-HDLCS technique is to recognize the presence of DDoS attacks, in order to ensure cybersecurity. In the presented WSEO-HDLCS technique, the high-dimensionality data problem can be resolved by the use of a WSEO-based feature selection (WSEO-FS) approach. In addition, the WSEO-HDLCS technique employs a stacked deep autoencoder (SDAE) model for DDoS attack detection. Moreover, the gravitational search algorithm (GSA) is utilized for the optimal selection of the hyperparameters related to the SDAE model. The experimental evaluation of the WSEO-HDLCS algorithm is validated on the CICIDS-2017 database. The widespread simulation values highlighted the promising outcome of the WSEO-HDLCS method over existing approaches.

## 2. Related Works

Ali and Li [10] introduced an effective DDoS attack detection method that depends on multi-level AE-based feature learning. The authors learned of multiple levels of shallow and DAE in unsupervised learning that can be utilized for encoding the trained and test information in feature generation. The ultimate combined identification technique is learned by integrating multiple-level features utilizing an effective multiple kernel learning (MKL) method. Monday et al. [11] proposed a technique for detecting DDoS attacks on the SG framework. Continuous wavelet transform (CWT) has been employed in the proposed method to transform 1D traffic data to a 2D time-frequency domain scalogram as the input to a wavelet CNN (WavCovNet) for detecting anomalous performance, with information by differentiating attack features in standard outlines. Diaba and Elmusrati [12] suggested a hybrid DL approach, which focused on DDoS attacks on the transmission framework of SGs. The recommended technique is hybridized by the GRU and CNN methods. Nagaraj et al. [13] introduce graph learning techniques to identify and detect DDoS attacks in SDN_SGC systems (GLASS). Network model statistics have been applied to model SDN_SGC graphs that are trained GCN for extracting hidden representations caused by DDoS attacks.

Ebojoh and Yeboah-Ofori [14] introduced an agent-based model of offensive botnet connections in an SG method, and studied the amplification attack strategy of FDIA and DDoS on SGs. Primarily, the authors examine that botnet agent attacks methods utilizing ABS influence collaborative protection in FDIA and DDoS attacks. Secondarily, the authors implemented an attack model utilizing the GAMA tool for determining offensive botnet interactions within an SG system. Lastly, the authors suggested control methods for preventing offensive botnets on the SG network. In [15], a model that depends on ML to identify SG DDoS attacks was suggested. The model initially gathers network information, then FS, applies PCA for reducing the data size and, lastly, utilizes the SVM approach to detect the abnormality.

Ma et al. [16] recommended an innovative DDoS attack identification technique that only applies unlabeled abnormal network traffic information to make the recognition system. This approach primarily utilizes the Balanced Iterative Reducing and Clustering utilizing the Hierarchies technique (BIRCH) for pre-clustering the anomalous network traffic data and, after examining AE, to make the identification method in unsupervised learning depends on clustering subsets. Khoei et al. [17] present a CNN-based approach, a ResNEt with 50 layers. In this method, the tabular information is modified to images for enhancing the model performance.

## 3. The Proposed Model

**Figure 1:**
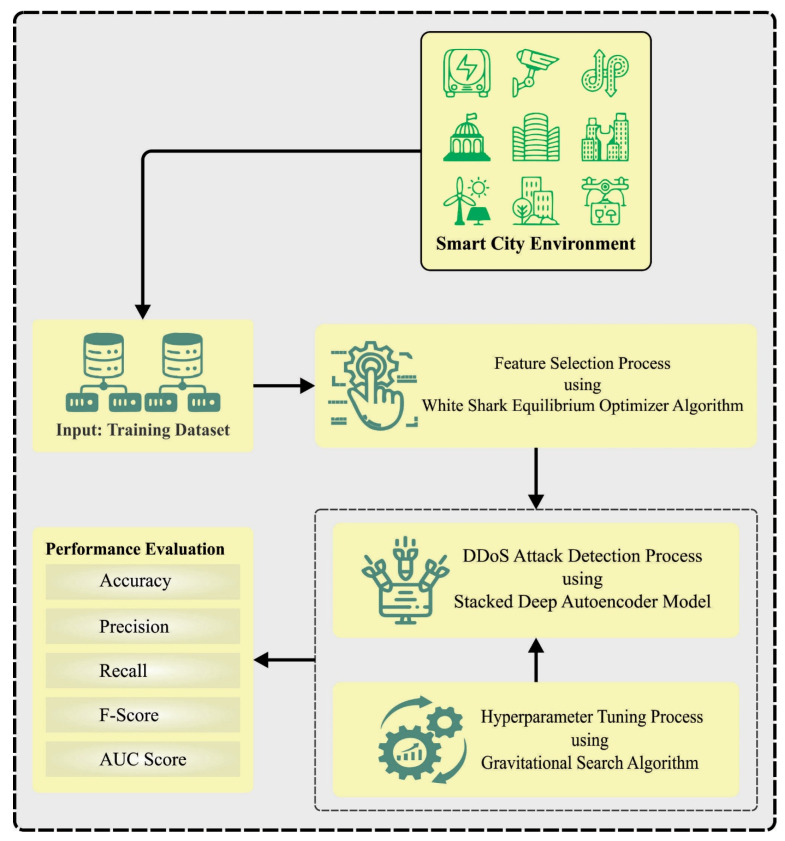
Overall flow of WSEO-HDLCS system.

In this study, we have designed and developed a WSEO-HDLCS methodology for cybersecurity in an SG environment. The major purpose of the WSEO-HDLCS system is to recognize the presence of DDoS attacks, in order to ensure cybersecurity. In the proposed WSEO-HDLCS system, three main sub-processes are contained in the WSEO-FS technique, SDAE-based classification, and GSA-based hyperparameter selection. Figure 1 exemplifies the overall flow of the WSEO-HDLCS method.

### 3.1. Design of WSEO-FS Technique

To choose a subset of features, the WSEO-FS technique is used. The WESO algorithm is derived by the use of a White Shark Optimizer (WSO) with an equilibrium optimizer (EO) [18]. In this work, the EO was used to increase the population of the worse solution and improve the WSO’s searching abilities. Due to its higher performance, the EO is applied to deeply search in the rugged search space by maintaining the balance among local as well as global searches. The study implements the EO to improve the worse solution by arranging the population and allowing for the second half as its population. The EO enhances the worse half of the population and returns it to the WSO for re-evaluating the population and selecting the better solution.

Initialization of parameters  WSO and EO: This step is used for initializing the WSO and EO parameters. For EO, the parameters are GP and V. For the *WSO*, the parameters are v, u, l, $\tau ,{\;f}_{min},{\;f}_{min},$ $p_{min}$, and $p_{max}.$

Initially, the initial population is produced. The population is randomly produced similar to other swarm-based optimizers, which consider the starting time st and the number of SAs (m).

(1) $$Population=\left[\begin{array}{cccc} st_{1}^{1} & st_{1}^{2} & \ldots & st_{m}^{1}\\ st_{2}^{1} & st_{2}^{2} & \ldots & st_{m}^{2}\\ \vdots & \vdots & \ldots & \vdots \\ st_{1}^{N} & st_{2}^{N} & \ldots & st_{m}^{N} \end{array}\right],\;$$

Next, the fitness value (FV) of the solution is assessed. Consequently, the *WSO* assigned the fittest outcome with the best values to $\omega_{gbest}.\;$ The searching agent of the *WSO* is used for updating the solution from the population and searching for the best schedule for the FS. Once it evaluates the FV for each solution from the population and allocates the fittest outcome to $\omega_{gbest}$, the *WSO* operation can upgrade and produce novel solutions based on the $\omega_{gbest}$. If they have optimum FV, then a new solution will replace the worst solution. Next, based on the FV, the solution from the population was ranked, where the best solution was highly ranked, and the worst solution was lowly ranked. After ranking the solution, the *EO* takes the solution with the low rank from the *WSO* population for additional improvement. The low-ranking solution is utilized as an initial population for the EO. The *EO* allocates the fittest four solutions to ${\vec{C}}_{eq(1)},$ ${\vec{C}}_{eq(2)},$ ${\vec{C}}_{eq(3)},$ and ${\vec{C}}_{eq(4)}$ for generating ${\vec{C}}_{eq.pool}$. Consequently, the *EO* updates the population to enhance the FV and search for the best schedule. Consequently, the *EO* returns the novel solution to the *WSO* population. The fitness function (FF) assumes the classifier accuracy and the FS counts. It maximizes the classifier accuracy and minimizes the fixed size of FSs. Then, the following FF can be employed for measuring individual performances, as expressed in Equation (2).

(2) $$Fitness=\alpha *\;ErrorRate+\left(1-\alpha \right)*\frac{\#SF}{\#All\_F}\;$$

whereas ErrorRate stands for the classifier rate of errors employing the FSs. ErrorRate denotes the measured percentage of incorrect classification to the count of classifiers made, expressed as a value among zero and one. #SF refers to the count of FSs and #All_F denotes the entire count of elements from the original database. α is utilized for controlling the impact of classifier quality and subset length.

### 3.2. Design of SDAE Classifier

**Figure 2:**
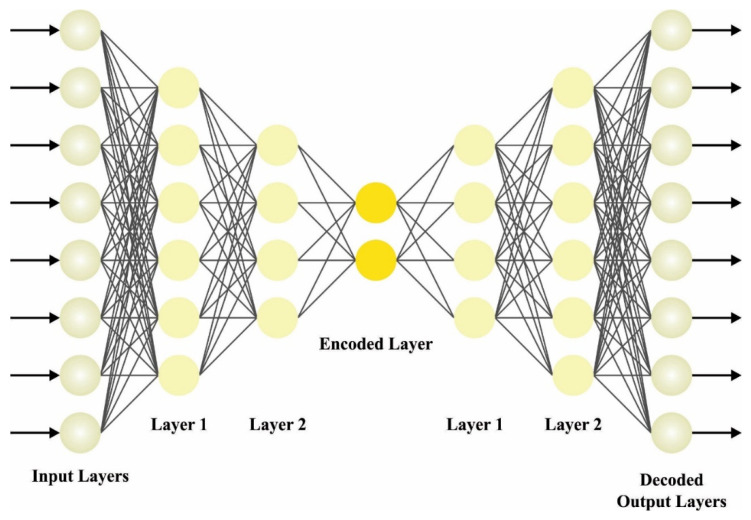
Architecture of SDAE.

For the identification of DDoS attacks, the SADE model is applied. In DAE, any trained parameters can be employed and written as the input vector $x_{i}(1,\;2,\;\ldots ,\;N)$ and as the hidden state $h_{i}$ [19]. An input vector calculates $x_{i}$ and a joint probability distribution function of $h_{i}$. It can be employed as the matrix weighted on the primary phase. Figure 2 portrays the infrastructure of SDAE. The estimate of the probability distribution function is provided as:

(3) $$p\left(h_{i}=1|x\right)=\sigma \left(b_{i}+\sum_{j}w_{ij}x_{i}\right)$$

whereas σ denotes the sigmoid function. The sigmoid function was determined as:

(4) $$\sigma =\frac{1}{1+e^{-z}}$$

The input data to a network can be provided as z, and the resultant data of the network are provided as $h_{w,b}(z)$, and $w_{ij}\left(i,\;j=1,\;2,\ldots ,\;N\right)$ signifies the primary weighted data. An input data point z can be stimulated using the mapping function to offer $m_{f}$ as:

(5) $$mf=sigm\left(w_{i}z+b_{i}\right)$$

in which sigm refers to the activation function, recognized as a sigmoid function:

(6) $$sigm=\frac{1}{1+e^{-z}}$$

The reconstructed signal in the decoded phase is expressed as:

(7) $${\hat{x}}_{i}=h_{w,d}\left(z\right)=g\left(w_{i}^{t}m_{f}+b_{i+1}\right)$$

The abovementioned formula, the weighted matrix and bias amongst the states (hidden and output) are defined as w and b.

The resultant features X attained later, and the decoded and the input data x attained before the encoded features, are the most important conditions of AE, and where the error appeared, reconstruction is provided by probability function:

(8) $$l(x,\;\hat{x})=\frac{1}{2}|x_{i}-{\hat{x}}_{i}{|}^{2}$$

SDAE contains several layers of encoded and decoded features, generating a deeper network. All layers of the encoded features decrease the data size, and all the layers of decoded features gradually reform the data back to its original size. The intermediate layer procedure is the compressed representations, and it develops gradually towards abstraction as it keeps moving deeper into a network.

The training of a SDAE is normally performed in a layer-by-layer method. It contains all the layers trained separately as AEs first. If the lower layers can be trained, they can be integrated as a single network and more fine-tuned as an end-to-end method.

### 3.3. Process Involved in GSA-Based Hyperparameter Tuning

Finally, the hyperparameters of the GSA model can be chosen by the use of GSAs. The GSA is inspired by the optimization strategy improved by the law of gravity [20]. In this technique, particles represent the object, while masses are used for the performance measurement. The particles communicated by using the laws of action and Newton’s law of gravity. Consider a solution that contains N particles (masses).

(9) $$x_{i}=\left(x_{i}^{1},\ldots \ldots x_{i}^{d}\ldots \ldots x_{i}^{D}\right)\;for\;i=1,2,3\ldots .n\;$$

In Equation (9), $x_{i}^{d}$ indicates the position of particle i at d dimension, and D denotes the overall amount of dimensions. All the performances of the particles are defined by the mass and measured by a vigor process. The gravity and inertial masses of each particle were modernized and equalized with all the iterations:

(10) $$.M_{ai}=M_{pi}=M_{ii}=M_{i}$$

(11) $$m_{i}=\frac{fit_{i}-worst}{best-worst}$$

(12) $$M_{i}=\frac{m_{i}}{\sum_{j=1}^{N}m_{j}}$$

where $fit_{i}$ shows the $i^{th}$ particle FV, and best and worst denotes the particles’ highest and lowest fitness scores.

Maximization challenges are characterized as follows:

(13) $$best=\underset{\mathrm{j\epsilon}\{1,\ldots \ldots N\}}{\max}fit_{j}$$

(14) $$worst=\underset{\mathrm{j\epsilon}\{1,\ldots \ldots N\}}{\min}fit_{j}$$

Considering the reducing issues, which are different and are evaluated as follows:

(15) $$best=\underset{\mathrm{j\epsilon}\{1,\ldots \ldots N\}}{\min}fit_{j}$$

(16) $$worst=\underset{\mathrm{j\epsilon}\{1,\ldots \ldots N\}}{\max}fit_{j}$$

The gravity $F_{ij}^{d}$ exerted on $i^{th}$ particles from $j^{th}$ particles is computed using Equation (17):

(17) $$F_{ij}^{d}=G\frac{M_{pi}\times M_{aj}}{R_{ij}+\varepsilon }\times \left(x_{j}^{d}-x_{i}^{d}\right)$$

where ${\;M}_{aj}$ shows the kinetic gravity energy of $j^{th}$ particles and $M_{pi}$ is the sedentary gravity potential of $i^{th}$ particles. ε denotes the teeny invariant. G is designated the gravity acceleration. $R_{ij}$ shows the Euclidean space within two particles,

(18) $$G=G_{0}e^{-\alpha \frac{t}{T}}$$

In Equation (18), $G_{O}$ and α are adjusted initially and gradually decreased to control the search accuracy, T shows the max iteration. The force used on $i^{th}$ particles in d size is a random weight matrix of other gravitational forces of the particles.

(19) $$F_{i}^{d}=\sum_{j\in Kbest,j\neq i}rrand_{j}F_{ij}^{d}\;$$

where $rrand_{j}$ shows the constant random parameter within [0, 1]. During the search process, keeping equilibrium is crucial to avoid becoming trapped in the local optimal and to strike the symmetry within exploitation and exploration. Solely, particles Kbest with the most important fitness weights are used to have a gravitational attraction on another particle.

(20) $$Kbest=N\times \frac{per+(1-\frac{t}{T})\times (100-per)}{100}\;$$

where per represents the particles’ proportion that efficiently contributes towards different particles in the final analysis. The rate of $i^{th}$ particles in d size at t iteration can be defined as follows:

(21) $$a_{i}^{d}=\frac{F_{i}^{d}}{M_{ii}}\;$$

Now, $M_{ii}$ shows inertial mass of the $i^{th}$ particles. The velocity of the particle at d dimension is the proportion of current speed and velocity.

(22) $$v_{i}^{d}=rand_{i}\times v_{i}^{d}+a_{i}^{d}\;$$

Now, $rand_{i}$ denotes the invariant arbitrary variable within $\left[0,1\right]$ and provides the search for the random characteristic. In addition, the following equations evaluate the next location of the particles in dimension d.

(23) $$x_{i}^{d}=x_{i}^{d}+v_{i}^{d}$$

Fitness choice is a key aspect of the GSA system. Solution encoding can be utilized to assess a better solution for candidate performances. In this work, maximum accuracy can be considered as the fitness function, as given below.

(24) $$Fitness=max\left(P\right)$$

(25) $$P=\frac{TP}{TP+FP}$$

in which FP and TP imply the false and true positive values.

## 4. Results Analysis

In this study, the DDoS attack detection performance can be validated using the CICIDS-2017 dataset [21]. It holds 113,270 samples with two classes, as represented in Table 1.

**Figure 3:**
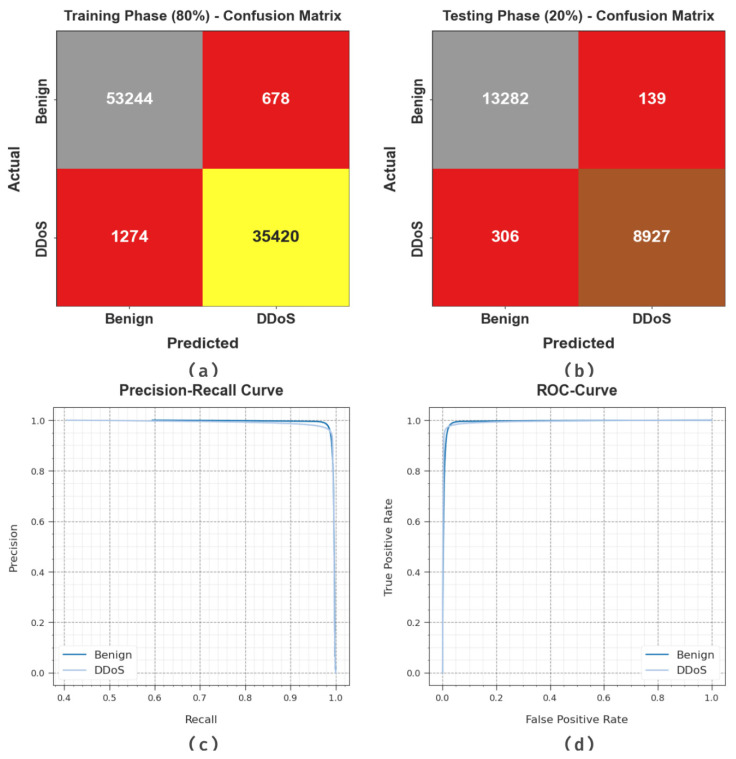
Performance of (**a**,**b**) 80% of TR set/20% of TS set, (**c**) PR_curve, and (**d**) ROC_curve.

Figure 3 reveals the classifier outcome of the WSEO-HDLCS algorithm on the test dataset. Figure 3a portrays the confusion matrix attained by the WSEO-HDLCS system on 80% of the TR set. The outcome inferred that the WSEO-HDLCS system has recognized 53,244 instances under the benign class and 35,420 instances under the DDoS class. Moreover, Figure 3b exemplifies the confusion matrix attained by the WSEO-HDLCS system on 20% of the TS set. The results signified that the WSEO-HDLCS methodology has recognized 13,282 instances under the benign class and 8927 instances under the DDoS class. Following this, Figure 3c represents the PR curve of the WSEO-HDLCS system. The outcome inferred that the WSEO-HDLCS system has achieved greater PR outcomes in two classes. But Figure 3d displays the ROC curve of the WSEO-HDLCS system. The result outperformed that the WSEO-HDLCS approach has led to capable performances with enhanced ROC values on two class labels.

**Figure 4:**
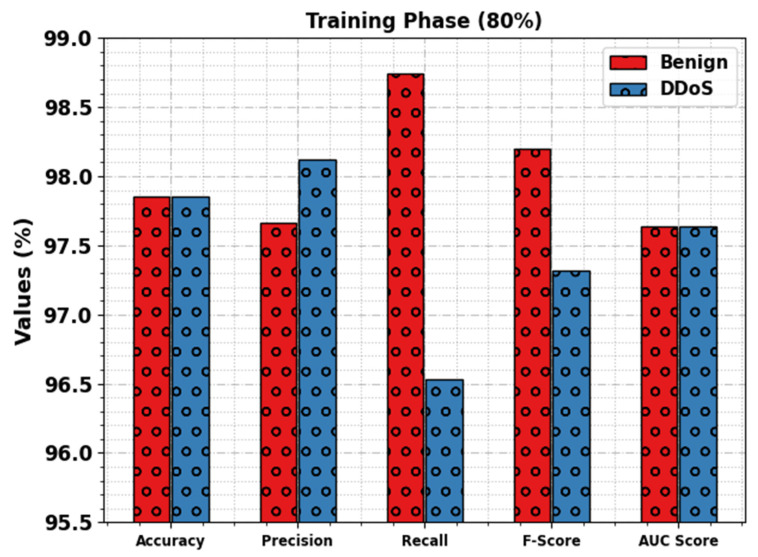
DDoS attack detection outcomes of WSEO-HDLCS approach on 80% of TR set.

Table 2 represents the DDoS attack detection results of the WSEO-HDLCS technique. Figure 4 inspects the overall results of the WSEO-HDLCS technique with 80% of the TR set. The outcomes inferred that the WSEO-HDLCS technique reaches enhanced identification of attacks. With the benign class, the WSEO-HDLCS technique offers $accu_{y}$, $prec_{n}$, $reca_{l}$, $F_{score}$, and $AUC_{score}\;$ values of 97.85%, 97.66%, 98.74%, 98.20%, and 97.64%, respectively. Additionally, with the DDoS class, the WSEO-HDLCS approach attains $accu_{y}$, $prec_{n}$, $reca_{l}$, $F_{score}$, and $AUC_{score}\;$ values of 97.85%, 98.12%, 96.53%, 97.32%, and 97.64%, respectively.

**Figure 5:**
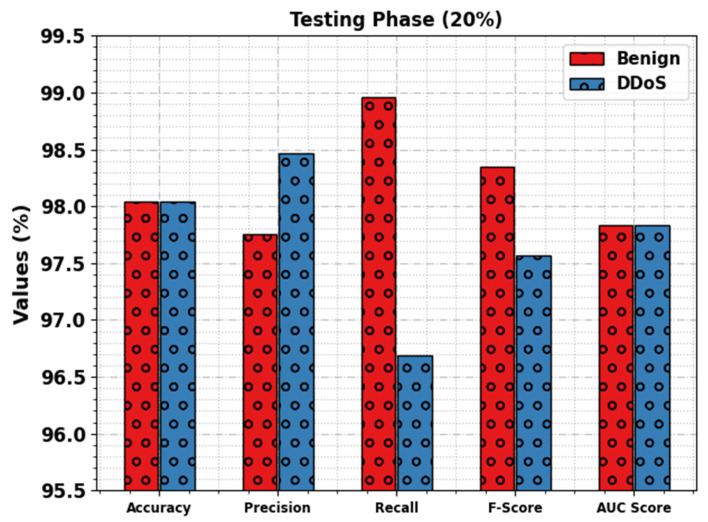
DDoS attack detection outcomes of WSEO-HDLCS approach on 20% of TS set.

Figure 5 examines the overall outcomes of the WSEO-HDLCS methodology with 20% of the TS set. The outcome inferred that the WSEO-HDLCS algorithm gains improved recognition of attacks. With the benign class, the WSEO-HDLCS methodology provides $accu_{y}$, $prec_{n}$, $reca_{l}$, $F_{score}$, and $AUC_{score}\;$ values of 98.04%, 97.75%, 98.96%, 98.35%, and 97.83%, respectively. Moreover, with the DDoS class, the WSEO-HDLCS methodology achieves $accu_{y}$, $prec_{n}$, $reca_{l}$, $F_{score}$, and $AUC_{score}\;$ values of 98.04%, 98.47%, 96.69%, 97.57%, and 97.83%, respectively.

**Figure 6:**
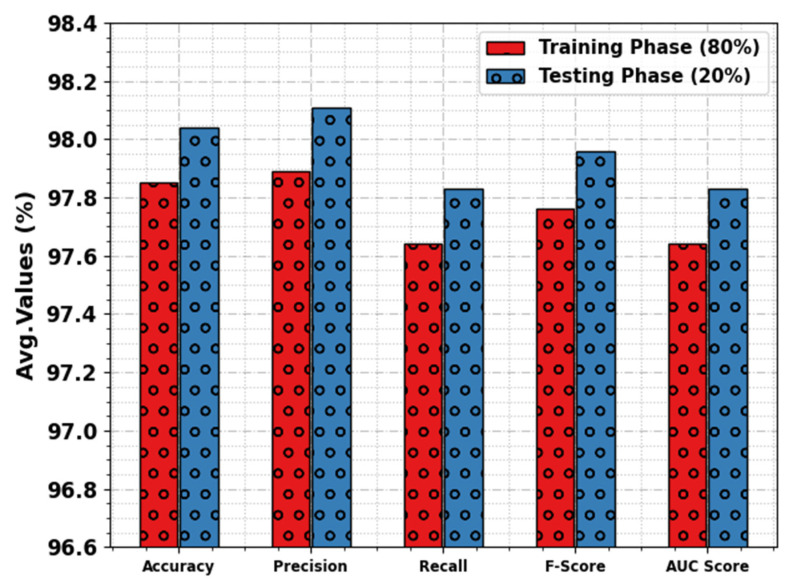
Average outcome of WSEO-HDLCS approach on 80% of TR set/20% of TS set.

Figure 6 inspects the overall average result of the WSEO-HDLCS algorithm with 80% of the TR set and 20% of the TS set. The simulation outcome denoted that the WSEO-HDLCS system gains greater detection of attacks. On 80% of the TR set, the WSEO-HDLCS method achieves average $accu_{y}$, $prec_{n}$, $reca_{l}$, $F_{score}$, and $AUC_{score}\;$ values of 97.85%, 97.89%, 97.64%, 97.76%, and 97.64%, respectively. On 20% of TS set, the WSEO-HDLCS algorithm reaches average $accu_{y}$, $prec_{n}$, $reca_{l}$, $F_{score}$, and $AUC_{score}\;$ values of 98.04%, 98.11%, 97.83%, 97.96%, and 97.83%, respectively.

**Figure 7:**
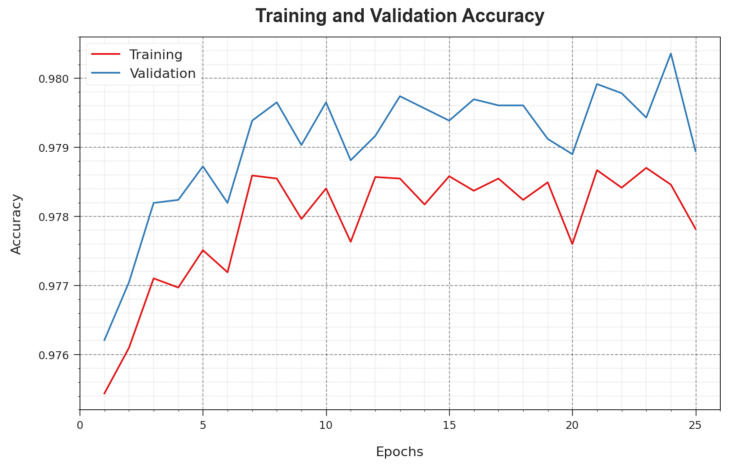
$Accu_{y}$ curve of the WSEO-HDLCS approach.

Figure 7 illustrates the training accuracy $TR\_accu_{y}$ and $VL\_accu_{y}$ of the WSEO-HDLCS approach. The $TL\_accu_{y}$ is defined by the assessment of the WSEO-HDLCS system on the TR dataset, whereas the $VL\_accu_{y}$ is calculated by estimating the solution on a separate testing dataset. The outcomes display that $TR\_accu_{y}$ and $VL\_accu_{y}$ enhance with an increase in epochs. Thus, the outcome the WSEO-HDLCS system obtains is greater on the TR and TS dataset with a rise in the count of epochs.

**Figure 8:**
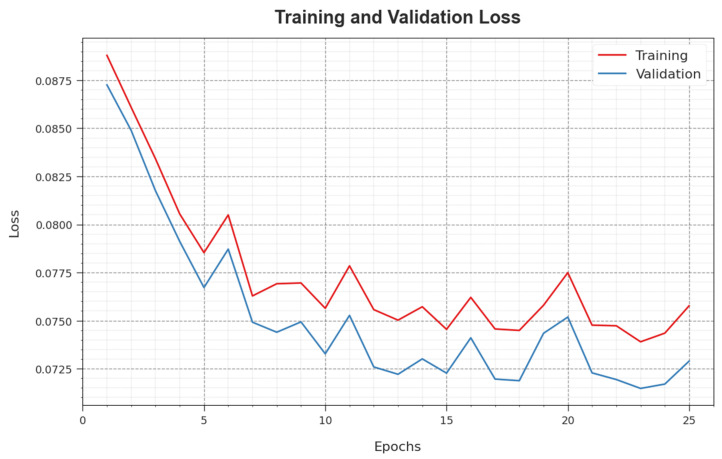
Loss curve of the WSEO-HDLCS approach.

In Figure 8, the TR_loss and VR_loss  curves of the WSEO-HDLCS system are exposed. The TR_loss demonstrates the error among the predictive solution and original values on the TR data. The VR_loss signifies the evaluation of the performance of the WSEO-HDLCS technique on individual validation data. The outcomes point out that the TR_loss and VR_loss tend to be less with increasing epochs. It represented the improved solution of the WSEO-HDLCS technique and its ability to produce an accurate classification. The minimal value of TR_loss and VR_loss reveals the improved outcome of the WSEO-HDLCS method on capturing patterns and relationships.

**Figure 9:**
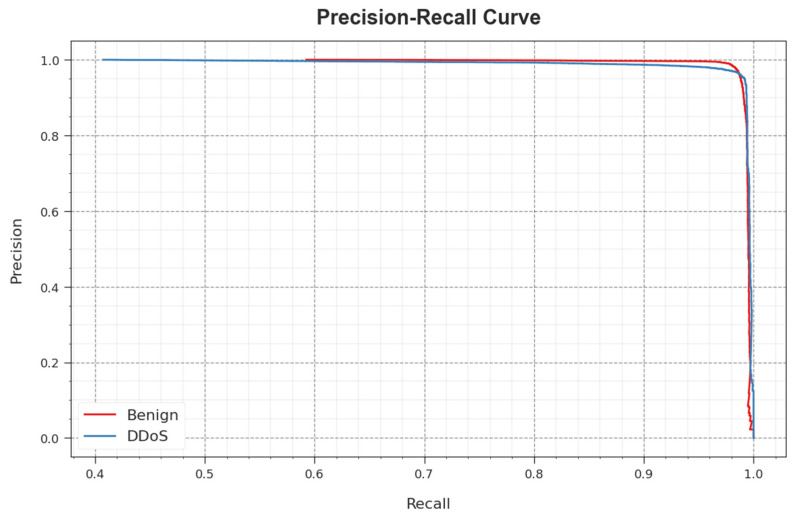
PR curve of the WSEO-HDLCS approach.

A comprehensive PR analysis of the WSEO-HDLCS algorithm is depicted on the test database in Figure 9. The simulation outcome inferred that the WSEO-HDLCS system outcomes enhanced the values of PR. Furthermore, it could be noticed that the WSEO-HDLCS algorithm attains greater PR values on two classes.

**Figure 10:**
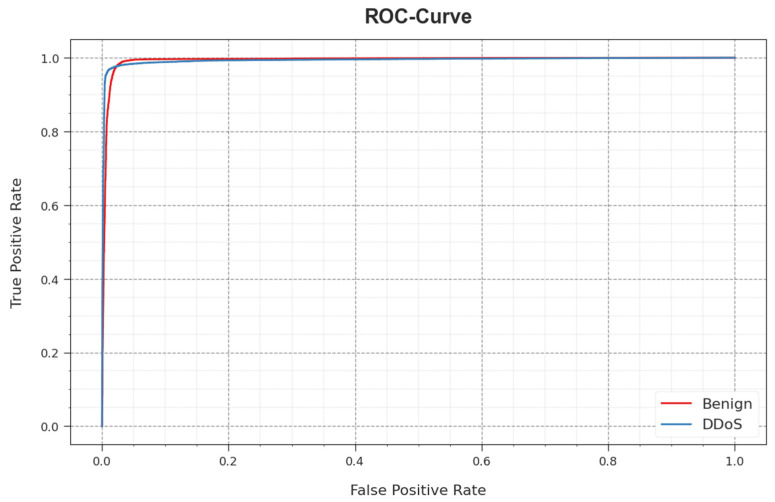
ROC curve of the WSEO-HDLCS algorithm.

In Figure 10, a ROC curve for the WSEO-HDLCS methodology on the test database is shown. The simulation value explained that the WSEO-HDLCS system gives rise to increased ROC values. Also, it can be observed that the WSEO-HDLCS algorithm extends greater ROC values on two classes.

**Figure 11:**
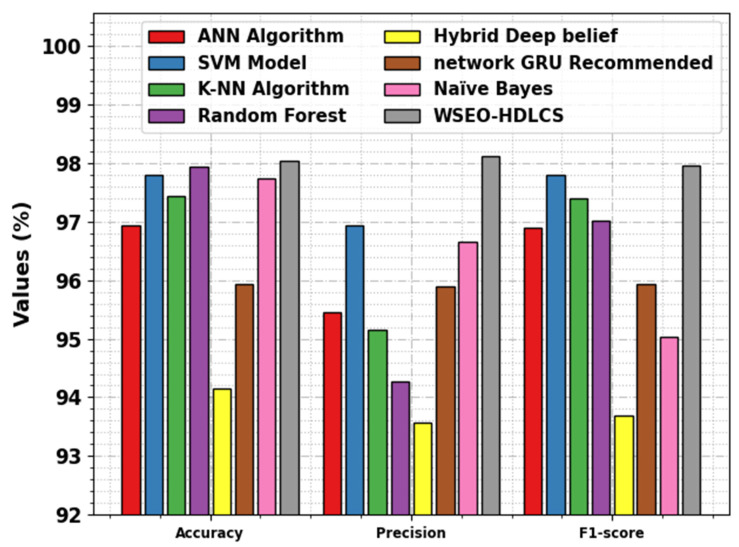
Comparative outcomes of WSEO-HDLCS approach with other methods.

Finally, the improved performance of the WSEO-HDLCS technique can be ensured by studying the comparisons in Table 3 and Figure 11 [12,22,23,24]. The simulation values portrayed that the hybrid deep belief and network GRU-recommended models have shown poor performance. Along with that, the ANN, SVM, KNN, RF, and NB approaches have reported moderate solutions.

Nevertheless, the WSEO-HDLCS algorithm exhibited superior performance, with a maximum $accu_{y}$ of 98.04%, $prec_{n}$ of 98.11%, and $F_{score}$ of 97.96%. These results confirmed that the WSEO-HDLCS technique can identify the DDoS attacks in the SG effectually.

## 5. Conclusions

In this manuscript, we have designed and established a WSEO-HDLCS algorithm for cybersecurity in the SG environment. The major purpose of the WSEO-HDLCS technique is to recognize the presence of DDoS attacks, in order to ensure cybersecurity. In the proposed WSEO-HDLCS system, the three main sub-processes contained are the WSEO-FS technique, SDAE-based classification, and GSA-based hyperparameter selection. The GSA is utilized for the optimal selection of the hyperparameters related to the SDAE model. The simulation value of the WSEO-HDLCS system was validated on the CICIDS-2017 database. The widespread simulation outcome highlighted the promising solution of the WSEO-HDLCS approach, compared to other methods. The proposed model offers various benefits in real-time applications, such as enhanced network resilience, reduced downtime, less service disruptions, reduced economic loss, effective resource utilization, and resilience against evolving threats. In future, the adaptability of the proposed model can be improved on evolving attacks using ensemble models. Additionally, real-time monitoring can be developed for the detection of DDoS attacks promptly. In addition, automated systems can trigger alarms or mitigation actions when suspicious traffic patterns are detected. Finally, flow-based monitoring and analysis approaches can be developed to gain insights into traffic flows, recognize suspicious activity, and isolate the sources of DDoS attacks.

## Figures and Tables

**Table 1:** Description of database.

Class	No. of Samples
BENIGN	67,343
DDoS	45,927
Total Samples	113,270

**Table 2:** DDoS attack detection outcome of WSEO-HDLCS approach on 80% of TR set/20% of TS set.

Class	$Accu_{y}$	$Prec_{n}$	$Reca_{l}$	$F_{Score}$	$AUC_{Score}$
Training Phase (80%)					
Benign	97.85	97.66	98.74	98.20	97.64
DDoS	97.85	98.12	96.53	97.32	97.64
Average	97.85	97.89	97.64	97.76	97.64
Testing Phase (20%)					
Benign	98.04	97.75	98.96	98.35	97.83
DDoS	98.04	98.47	96.69	97.57	97.83
Average	98.04	98.11	97.83	97.96	97.83

**Table 3:** Comparative outcome of WSEO-HDLCS approach with other methods.

Algorithms	$Accu_{y}$	$Prec_{n}$	$F1_{score}$
ANN Algorithm [22]	96.94	95.45	96.90
SVM Model [22]	97.80	96.93	97.80
K-NN Algorithm [22]	97.44	95.16	97.40
Random Forest [22]	97.94	94.28	97.01
Hybrid Deep belief [23]	94.14	93.57	93.68
Network GRU Recommended [12]	95.93	95.89	95.94
Naïve Bayes [24]	97.74	96.65	95.04
WSEO-HDLCS	98.04	98.11	97.96

## Data Availability

Data sharing is not applicable to this article, as no datasets were generated during the current study.
